# Reduced Frequency of Knowledge of Results Enhances Acquisition of Skills in Rats as in Humans

**DOI:** 10.3389/fpsyg.2020.00846

**Published:** 2020-04-30

**Authors:** Alliston K. Reid, Paige G. Bolton Swafford

**Affiliations:** Department of Psychology, Wofford College, Spartanburg, SC, United States

**Keywords:** skill learning, knowledge of results, operant feedback, frequency of KR, autonomy, comparative psychology, Rats, Humans

## Abstract

Macphail’s (1985) null hypothesis challenged researchers to demonstrate any differences in intelligence between vertebrate species. Rather than focus on differences, we asked whether rats would show the same unexpected, counterintuitive features of skill learning observed in humans: Factors that degrade performance during acquisition often enhance performance in a subsequent retention/autonomy phase. Providing post-trial “knowledge of results” (KR) on 30–67% of trials instead of 100% degrades accuracy, yet increases retention in a subsequent phase without KR. We tested this feature by providing three groups of rats with KR on every trial (100% KR), 67% KR, or 0% KR. We also provided operant feedback in every trial for completing the left-right lever-press skill (food for correct sequences, timeout for all others). In the autonomy phase, we assessed their ability to complete the skill independently—in the absence of differential cues and KR feedback. In agreement with human performance in the autonomy phase, 67% KR yielded higher skill accuracy than providing 100% KR. Also, providing 67% KR improved skill accuracy above that observed with operant feedback alone (0% KR). Rather than degrading performance during acquisition, the 67% KR condition yielded unexpected higher accuracy than the other conditions. Accuracy increased systematically across our extended acquisition phase, which provided each rat with over 3600 trials compared to 20–30 trials for human studies. Providing limited KR promoted skill learning in rats as it does in humans, consistent with the conjecture that both species share common learning processes. Introducing difficulties to rats during training improved their autonomy.

## Introduction

Skill learning has been widely studied in humans, but far less in rats. In the last few decades, researchers have often focused on a counterintuitive feature of skill learning in humans related to the historical distinction between learning and performance dating back to Thorndike’s (1927) law of effect. Empirical reviews by Schmidt and Bjork (1992), Soderstrom and Bjork (2015), and Johnson and Proctor (2017) have described this common feature across several motor- and verbal-learning paradigms: Factors that degrade performance during acquisition often enhance performance in a subsequent retention condition (see also Bjork and Bjork, 1992, 2014; Kantak and Winstein, 2012). Soderstrom and Bjork explained that introducing “desirable difficulties” (Bjork and Bjork, 2014) for the learner during acquisition can enhance later retention because the cognitive processes active during these “difficulties” link new information with knowledge that already exists in memory.

As implied above, skill-learning studies usually consist of two phases: an acquisition phase in which the skill is acquired in the presence of useful stimuli or feedback from performance, and a retention phase in which retention of the skill is measured when those cues and feedback are no longer provided. In studies with non-humans such as rats or pigeons, we usually call this retention phase an “autonomy” phase because we are interested in the degree to which the animal can complete the skill independently—in the absence of earlier guiding cues or informative feedback. Humans typically seek autonomy as we acquire behavioral skills related to sports, driving, military training, dance, our jobs, and games of skill. Applied behavior analysis often promotes skill learning and autonomy in children with developmental disabilities. Extensive practice of behavioral skills usually leads to autonomy in humans and trained animals, even rats and pigeons (e.g., Helton, 2007a,b; Reid et al., 2010, 2013a).

Behavioral skill learning (as distinguished from cognitive skills such as playing chess) could focus on the roles of (a) antecedent stimuli, or on (b) feedback from performance. Rat studies have focused on anticipatory cues, such as transfer of stimulus control from discriminative stimuli to new exteroceptive or endogenous cues that develop during practice. For example, Mackintosh (1965, 1974) described examples of transfer to proprioceptive control as rats run through mazes and suggested that “if sufficient training were given on a maze problem, control was gradually transferred from exteroceptive to proprioceptive stimuli” (1974, p. 554). Several rat and pigeon studies have observed the same counterintuitive feature of human skill learning: *Less effective* cues and *more difficult* behavioral skills degrade accuracy during acquisition phases, yet *enhance* accuracy in a subsequent autonomy phase (e.g., Rats: Reid et al., 2010, 2013a,b, 2014, 2017; Pigeons: Fox et al., 2014). We often described these observations with the metaphor: “Holding your child’s hand too much may delay or prevent autonomy.”

In contrast, skill-learning studies with humans have historically focused on feedback from performance. While many types of feedback may be provided, the most influential type has been “knowledge of results” (KR): post-trial feedback about the success of a behavior during skill acquisition (Adams, 1987; Wulf and Schmidt, 1989; Newell, 1991; Wulf and Shea, 2002; Wolpert et al., 2011). One might predict that providing KR on every trial (100% KR) would lead to faster acquisition and stronger retention. However, Winstein and Schmidt (1990) demonstrated a counterintuitive feature of providing different relative frequencies of KR as feedback to human participants: Practice with reduced 50% KR improved skill retention over 100% KR, even though performance during acquisition suffered. This observation has been widely (although not universally) replicated with different types of skill learning in humans. Similarly, Wulf and Schmidt (1989) evaluated the effect of KR on a more complex behavioral skill (generalized motor programs) by comparing 67% KR versus 100% KR. Practice with reduced 67% KR was more beneficial to the transfer of skill learning even with this more complex behavioral skill. These studies were consistent with the claim (Schmidt and Bjork, 1992; Bjork and Bjork, 2014) that introducing difficulties for the learner during acquisition can enhance later retention.

Unfortunately, most human studies of skill learning ignore the other type of feedback: the operant consequences of responding such as reinforcement and punishment effects. Studies with rats or pigeons consistently specify these operant consequences. To our knowledge, no published rat or pigeon studies have included KR feedback with operant feedback. Therefore, it is unknown whether rats and humans would respond similarly to acquisition procedures that provide KR feedback. If rats and humans show not only the same basic features of skill learning, but also the same counterintuitive features, this would seem like much stronger evidence that skill learning in rats and humans may involve similar processes. Therefore, the purpose of this study was to begin to answer that very question.

This experiment combined operant feedback with KR feedback, so it is helpful to clarify the difference between the procedures, as well as the ways in which each affects skill acquisition and retention. However, our understanding of both terms and their mechanisms of action have changed substantially over the last century. Thorndike (1927) considered both types of post-trial feedback to be consistent with his Law of Effect: food delivery and KR indicating performance successes would both strengthen the stimulus-response connection, whereas extinction and KR indicating performance errors would weaken the connection. This view changed substantially after Salmoni et al. (1984) reviewed many studies of skill learning that provided KR feedback to human participants and asked: How does KR work? They emphasized the surprising observation that reduced frequencies of KR typically led to greater retention of motor skills than when KR was presented on every trial. Their “guidance hypothesis” emphasized the informative, goal-directed properties of KR feedback, which was more compatible with information-processing views of human cognition than the reinforcing properties of feedback. Today, KR in human studies is generally believed to provide opportunities to allocate cognitive resources such as attention and encoding processes, or to effectively link new information with existing memory. Several articles have expressed doubts as to whether laboratory animals would have the information-processing capacities to benefit from KR feedback (e.g., Adams, 1987; Wulf and Schmidt, 1989; Wulf and Shea, 2002). This claim assumes that Macphail’s’s (1985) null hypothesis is wrong, but (until now) no studies with laboratory animals have tested the assumption that laboratory animals and humans would react differently to skill learning procedures that provide KR.

Our behavioral skill was a simple left-right (L-R) lever-press sequence guided by blue and green panel lights above the two levers. All other sequences were errors. The second press terminated the trial and provided both operant and KR feedback. Operant feedback was always immediate and preceded KR feedback. Following the KR procedures of Wulf and Schmidt (1989) in a between-groups design, we compared the effects of 67% KR with those of 100% KR. As a control condition, we also included a 0% KR group that received operant feedback every trial, but never KR feedback, in both acquisition and autonomy phases. Normally, pellet delivery and KR feedback would be perfectly correlated for the 100% KR group. To distinguish between these two factors, we degraded this correlation by providing reinforcement for correct trials on a random ratio 2 (RR-2) schedule of reinforcement. If KR affects rats in the same way as in humans, then sequence accuracy during the autonomy phase should be higher for the 67% KR group than the 100% KR group, but accuracy for the 67% KR group during the acquisition phase should be lower than the 100% KR group. If providing KR promotes skill learning beyond the effects of operant feedback alone, then accuracy for the 67% KR group should exceed that of the 0% KR group.

## Methods

### Subjects

Twenty-six naïve 4-month-old female Long Evans rats (*Rattus norvegicus*) were housed individually in a facility that maintained constant temperature and humidity on a 12:12-h light:dark cycle. Body weight was maintained at 80–85% of free-feeding weight by providing supplemental food (Tekland Rodent Diet) after daily sessions in home cages with water freely available.

### Apparatus

We utilized four standard Med Associates operant chambers for rats, measuring 30 cm × 24 cm × 22 cm. Each chamber was located inside an isolation chamber containing a ventilation fan and a 7-W nightlight. A sound generator produced constant 65-db white noise in each chamber. Each operant chamber contained two retractable levers on the front wall and two non-retractable levers on the rear wall. Each pair of levers was separated by 16.5 cm, center to center, and located 6 cm above the floor. The 5 cm × 5 cm magazine hopper was centered between the two response levers on the front wall, 3 cm above the floor. A 2.5-cm 28-V white panel lamp was located 2.5 cm above the two front levers, and a 28-V houselight was located at the center top of the rear wall. In some conditions, 28-V blue or green LEDs replaced the two white panel lamps on the rear wall. The wavelengths of these LEDs (blue: 465 nm, green: 515 nm) were selected to approximately match the peak photopigment sensitivity of the UV and M cones (358 and 510 nm, respectively) in Long-Evans rats (Jacobs et al., 2001). A Sonalert was available to produce 1000-Hz tones. The pellet dispenser provided 45-mg Research Diet pellets. All four chambers were controlled by a single Lenovo personal computer located in an adjacent room and programmed in MED-PC IV, which implemented all experimental conditions and recorded every event and time of occurrence with 10-ms resolution.

### Procedure

We randomly assigned the 26 naïve rats to three experimental groups of 8,9 rats each. The groups differed in the frequency of qualitative KR feedback about the accuracy of responding, which was provided by the two white front panel lamps and the Sonalert tone for responding on the two rear levers in a discrete-trials procedure. The 0% KR group received no KR on any trial; the 100% KR group received KR on every trial; and the 67% KR group received KR on 67% of the trials.

We exposed all three groups to a sequence of training procedures that reinforced lever pressing on the front wall. Once lever-press training was completed, the experiment consisted of two experimental phases on the rear wall. The purpose of the 30-session Acquisition Phase was to allow each group of rats to learn the left-right (L–R) lever-press sequence (the skill) on the rear wall, guided by blue (left) and green (right) LED panel lights. In the final 10-session Autonomy Phase, the reinforcement contingencies were unchanged, but KR was eliminated, and the rear blue and green LED panel lights were replaced with white LEDs. Our primary measure was percentage L–R accuracy, which allowed us to assess (a) the speed in which the three groups learned the L–R sequence during the Acquisition Phase, and (b) the degree of L–R autonomy once we eliminated the differential stimuli and KR in the Autonomy Phase.

#### Training

##### Lever-press training

We exposed all rats to an autoshaping procedure for three sessions. The procedure inserted the right front retractable lever (adjacent to the food hopper) into the chamber 8 s before delivering a pellet, followed by a 52-s intertrial interval (ITI). Each press on that lever or any of the three other levers was reinforced, independent of the 8-s lever insertion. Sessions ended with the earlier of 45 min or 80 pellets.

We next exposed each rat to a shaping procedure in which presses to the front right retractable lever or either rear lever produced pellet delivery. The white panel lamp over the front right lever was illuminated, but the rear panel lamps remained off. Pellet deliveries were followed by 3-s ITI, signaled by extinguishing that panel light and the houselight. Sessions ended with the earlier of 30 min or 45 pellets. This training procedure terminated when the rat earned 45 pellets/session for three sessions. All subsequent conditions required subjects to press levers on the rear wall, so levers on the front wall were retracted for the rest of the experiment.

##### Rear wall

We exposed each subject to training sessions of fixed ratio-1 (FR-1) for pressing the right lever on the rear wall. The green panel light above that lever was illuminated, while the other panel lamps remained extinguished. Food delivery was followed by a 3-s ITI, signaled by extinguishing the panel light and houselight. Sessions ended with the earlier of 30 min or 45 reinforcers. This training procedure terminated when the rat earned 45 pellets/session for three sessions.

Following this training, we exposed each subject to training sessions for pressing the left lever on the rear wall on FR-1. The blue panel light above that lever was illuminated, while the other panel lamps remained extinguished. Otherwise, conditions were the same as training on the right lever. The purpose of these rear-wall training sessions was to ensure all subjects received approximately equal exposure to the reinforcement conditions on the rear wall before the experiment began, given that subjects required differing amounts of lever-press training on the front wall.

#### Experiment: L–R Acquisition Phase

The Acquisition Phase lasted 30 sessions for all subjects. It required subjects to complete a L–R lever-press sequence guided by blue (left) and green (right) panel lights in a discrete-trials procedure. No feedback about response accuracy was provided until two lever presses occurred (correct or not), which always produced a 100-ms tone “beep” end-of-trial marker. At the beginning of each trial, the houselight and rear blue (left) and green (right) panel lights were illuminated. Pressing the left lever turned off the left panel light, leaving only the right (green) panel light on. Similarly, pressing the right lever first would turn off the right panel light, leaving only the left (blue) panel light on. This was intended to reduce perseveration on either lever. Completion of the correct L–R response sequence turned off the houselight and panel lights. In addition, a food pellet was delivered with probability 0.5, producing a random ratio 2 (RR-2) reinforcement schedule. The RR-2 schedule was intended to degrade the normal correlation in discrete-trials procedures containing both food delivery and KR feedback. Incorrect trials contained any other 2-response sequence (L–L, R–L, R–R) and resulted in a 3-s timeout (TO) in which the houselight and panel lights were extinguished. Responding during TO had no programmed consequences. The next trial began with the illumination of the houselight and blue and green panel lights. Sessions lasted for the earlier of 45 min or until 120 correct L–R trials occurred (producing approximately 60 pellets on the RR-2 schedule).

**FIGURE 1:**
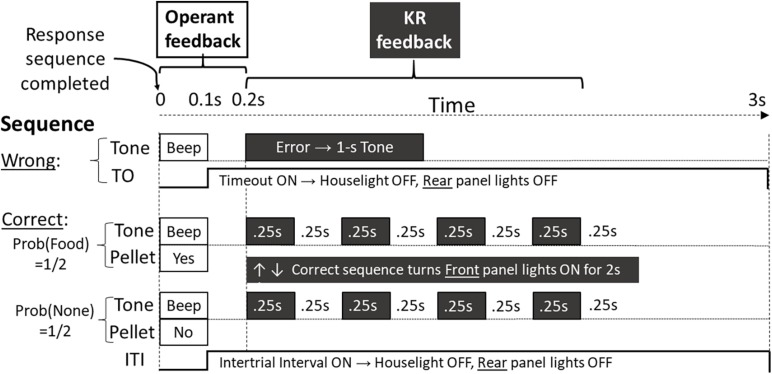
Timing diagram for operant feedback and KR feedback, depicted in different fonts. Operant feedback is depicted in black text with white background, whereas KR feedback is depicted in white text with black background. See procedure for other details.

Following the operant consequences described above, the three experimental groups received KR feedback delayed by 200 ms with probabilities identified by the name of each group: 0% KR, 67% KR, or 100% KR. As Figure 1 illustrates, the qualitative probabilistic KR feedback was different for correct trials and incorrect trials. Correct sequences produced KR in which both front white panel lights turned on for 2 s, while the Sonalert sounded four times in that 2-s interval (each 0.25-s on, 0.25-s off). Following incorrect sequences, the Sonalert sounded for a full second, and the front panel lights remained off.

#### Experiment: Autonomy Phase

The 10-session Autonomy Phase was identical to the Acquisition Phase except: (a) The blue and green LED panel lights were replaced by identical white LED lights, and (b) No KR feedback was provided in any trial to any group. The lamps functioned the same way within trials as before (e.g., during ITI and TO), but the white lights above both levers provided no discriminative cues to influence lever selection. The autonomy condition assessed how well each rat could complete the L–R sequence independently, without the differential cues provided by panel lights or potential influence of KR feedback.

## Results

**FIGURE 2:**
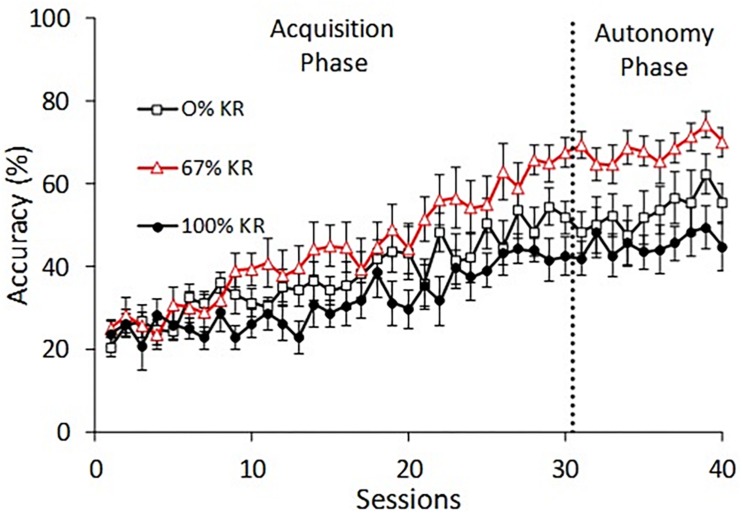
Percentage accuracy of the L-R behavioral skill for Groups 0% KR, 67% KR, and 100% KR across the 30 sessions of the Acquisition Phase and 10 sessions of the Autonomy Phase. Error bars depict standard error of the mean.

Figure 2 depicts L-R sequence accuracy for the three KR groups across the Acquisition and Autonomy Phases, separated by the vertical dotted line. We adopted an alpha level of.05 for all statistical tests.

### Acquisition Phase

We used a mixed-effects 2-way ANOVA across the 30 sessions of the Acquisition Phase to assess two potential main effects (group, sessions) and a potential group × sessions interaction. We observed a significant repeated-measures effect of sessions, *F*(29, 667) = 20.105, *p* < 0.001, η_p_^2^ = 0.446, confirming that accuracy generally increased across acquisition sessions. We also observed a statistically significant group × sessions interaction, indicating that some groups learned faster or slower than average, *F*_(58, 667)_ = 1.531, *p* = 0.008, η_p_^2^ = 0.118. This significant interaction should be expected to qualify any main effect of group. Even so, the ANOVA showed a nearly significant main effect of group, *F*_(2, 23)_ = 3.369, *p* = 0.052, η_p_^2^ = 0.227. An LSD *post hoc* test demonstrated that accuracy for the 67% KR group increased faster than for the 100% KR group, *p* = 0.016. There were no significant between-group differences during the Acquisition Phase between the 0% KR group and the 67% KR group, nor between the 0% KR group and the 100% KR group.

### Autonomy Phase

Figure 2 illustrates continued increasing accuracy through autonomy sessions. A mixed-effects 2-way ANOVA demonstrated a main effect of sessions in the Autonomy Phase, Wilks Lambda = 0.262, *F*(9, 15) = 4.698, *p* = 0.004, η_p_^2^ = 0.738, observed power = 0.964. The sessions × group interaction was not statistically significant, indicating the groups learned similarly during the Autonomy Phase, Wilks Lambda = 0.442, *F*_(18, 30)_ = 0.839, *p* = 0.646. The main effect of group was statistically significant, *F*(2, 23) = 7.112, *p* = 0.004, η_p_^2^ = 0.382, observed power = 0.895. The Autonomy Phase shows that L-R accuracy for the 67% KR group was significantly greater than the 100% KR group, as confirmed with the LSD *post hoc* test, *p* = 0.001. Similarly, the LSD *post hoc* test indicated that the 67% KR group achieved significantly higher accuracy during the Autonomy Phase than the 0% KR group, *p* = 0.033. The 0% KR group appears slightly higher than the 100% KR group, but the difference was not statistically significant, *p* = 0.19.

### Analysis of Errors

**FIGURE 3:**
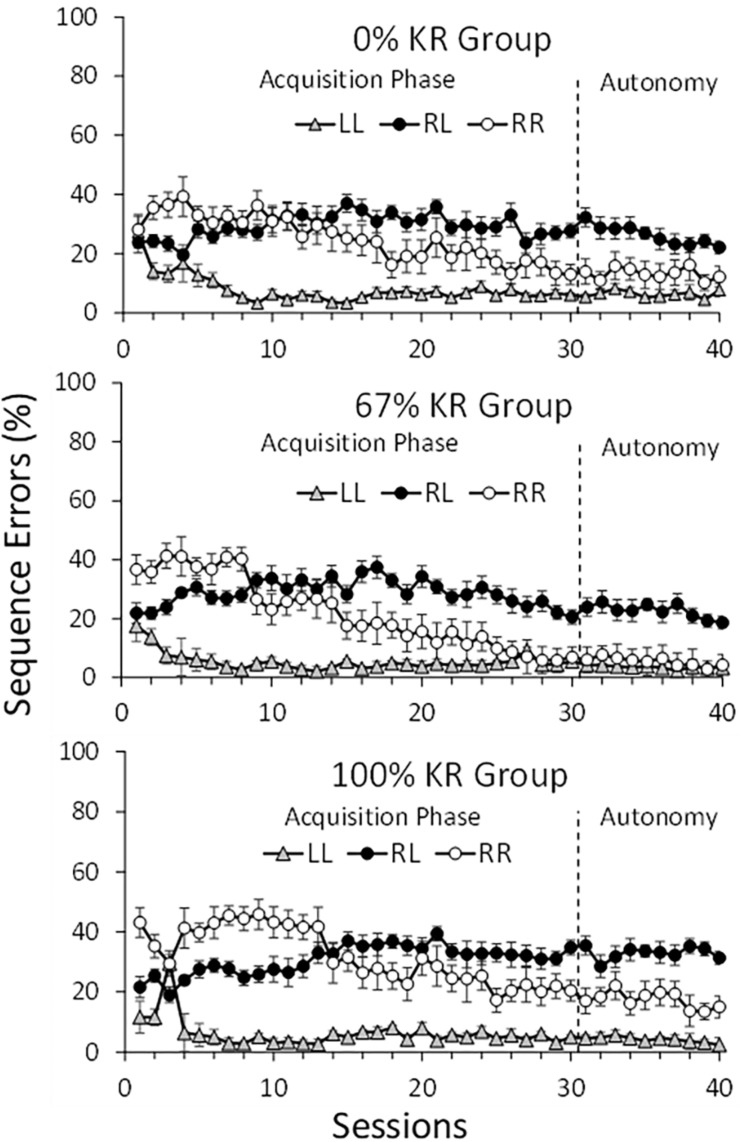
Percentages of the three possible response-sequence errors (LL, RL, RR) depicted for the three KR groups across the Acquisition and Autonomy Phases. Note that the patterns of errors were similar for the three KR groups across both phases. Error bars depict standard error of the mean.

Figure 3 identifies the frequencies of response-sequence errors (L-L, R-L, R-R) observed in the Acquisition and Autonomy Phases for each KR group. The patterns of these errors were remarkably consistent across the three groups.

## Discussion

The purpose of this study was to begin answering the question: Does KR affect rats the same way as with humans? Many studies have demonstrated the counterintuitive feature of human skill learning that practice with reduced relative frequency of KR improves skill retention over 100% KR, even though performance during acquisition often suffers (e.g., Wulf and Schmidt, 1989; Winstein and Schmidt, 1990; Schmidt and Bjork, 1992; Kantak and Winstein, 2012; Bjork and Bjork, 2014; Soderstrom and Bjork, 2015). Our rats demonstrated the same clear effect. Accuracy in the Autonomy Phase for the 67% KR group was greater than the 100% KR group: significantly greater accuracy with reduced KR feedback. The rats replicated the counterintuitive feature of skill learning that has come to define how motor skill learning occurs in humans. Yet performance during our Acquisition Phase did not suffer: Our 67% KR group yielded higher accuracy than the 100% KR group throughout both phases. This difference between rats and humans is surely due to the procedural differences in the number of trials experienced during acquisition. Human studies typically provide only about 20–30 acquisition trials (total), whereas our rats received an average of 3615 trials—more than 100 times as many (about 120 trials per session for 30 sessions). Performance in human KR studies improves for every group during acquisition phases (not only in the brief retention phase), consistent with the power law of practice. Therefore, one should expect improvement to continue when the duration of the Acquisition Phase is greatly extended, as in this experiment.

To our knowledge, this is the first experiment with rats that explicitly combined operant and KR feedback. By including a 0% KR group that never received any KR feedback, our procedure allowed us to measure whether the addition of KR feedback influences accuracy beyond that of operant feedback alone. Figure 2 shows that the 67% KR group produced significantly higher accuracy than the 0% KR group during the Autonomy Phase. Thus, providing 67% KR feedback did improve autonomy above that of operant feedback alone.

KR experiments with humans have repeatedly demonstrated that acquisition is improved by providing some (“but not too much”) KR feedback. However, they do not normally provide a 0% KR baseline condition for comparison. We observed that providing 67% KR was not “too much” compared to 0% KR because 67% KR was still beneficial. The differences between 100% KR and 0% KR were not statistically significant, but the differences were compatible with the claim that 100% KR was “too much” feedback to be beneficial for rats. An understanding of what entails “too much” feedback will require additional research. Nevertheless, our tentative observation is compatible with several hypotheses designed to explain how KR on every trial is less effective than less frequent KR, such as the guidance hypothesis (Salmoni et al., 1984) and explanations based on information processing. Of several theories attempting to explain this observation, Schmidt and Bjork (1992) suggested that frequent feedback in studies (with humans) could block important information processing activities from occurring during the acquisition phase that are required for learning. In contrast, when KR is given intermittently during the acquisition phase, human learners may be more able to evaluate performance in the absence of KR, and thus perform better in the retention phase (Winstein and Schmidt, 1990).

Schmidt and Bjork (1992) summarized procedures that share a common principle for learning motor and verbal skills in humans: “Introducing difficulties for the learner can enhance training” (p. 209). Providing reduced KR was one of these procedures, but others did not include KR. Bjork and Bjork (2014) recently described this principle as “Creating desirable difficulties to enhance learning” (p. 56). This principle also applies to acquisition of behavioral skills in rats and pigeons. As mentioned earlier, several rat and pigeon studies have observed the same counterintuitive feature of human skill learning: *Less effective* cues and *more difficult* behavioral skills degrade accuracy during acquisition phases, yet *enhance* accuracy in the subsequent autonomy phase (e.g., *Rats*: Reid et al., 2010, 2013a,b, 2017; *Pigeons*: Fox et al., 2014; Reid et al., 2014).

It is becoming increasingly apparent that skill learning in rats and humans share certain consistent features—even counterintuitive features. Some researchers may assume rats would not have the cognitive abilities to benefit from KR feedback (e.g., Wulf and Schmidt, 1989; Wulf and Shea, 2002) or that the mechanisms for learning in humans are not the same as those for animal conditioning with reinforcement (e.g., Winstein and Schmidt, 1990). Nevertheless, our rats replicated the major findings of KR procedures widely documented in human skill learning. The cognitive processes required for these procedures may be simpler than those proposed to explain results with humans. Identifying whether these similarities are due to common general principles of skill learning will require much more research, including explorations of the various forms of KR known to affect skill learning in humans (Winstein, 1991; Mazur, 2006). We believe this research will be worth the effort. We propose that experimental designs with animals (e.g., rats, pigeons, dogs) with clearly specified combinations of operant and KR feedback (e.g., Figure 1) have the potential to more clearly define the relationship between (and the importance of) these two classes of feedback that greatly influence both acquisition and retention in humans and laboratory animals. This discovery would be a substantial improvement in our understanding of skill acquisition across species.

## Data Availability Statement

The datasets generated for this study are available on request to the corresponding author.

## Ethics Statement

Ethical review and approval was not required for the study on human participants in accordance with the local legislation and institutional requirements. The patients/participants provided their written informed consent to participate in this study.

## Author Contributions

AR designed the experiment, obtained funding, programmed all conditions, helped analyse the data, and wrote the final manuscript. PS supervised the research team to carry out the experiment, helped with data analysis, and contributed to early versions of the manuscript.

## Conflict of Interest

The authors declare that the research was conducted in the absence of any commercial or financial relationships that could be construed as a potential conflict of interest.
